# Hyper-acidic fusion minipeptides escort the intrinsic antioxidative ability of the pattern recognition receptor CRP in non-animal organisms

**DOI:** 10.1038/s41598-019-39388-8

**Published:** 2019-02-28

**Authors:** Mengru Zhang, Yanjuan Liu, Zhibin Liu, Jianmei Wang, Ming Gong, Hu Ge, Xufeng Li, Yi Yang, Zhurong Zou

**Affiliations:** 1 Key Laboratory of Bio-Resources and Eco-Environment of Ministry of Education, College of Life Sciences, Sichuan University, Chengdu, 610064 China; 2 Engineering Research Center of Sustainable Development and Utilization of Biomass Energy, Ministry of Education, School of Life Sciences, Yunnan Normal University, Kunming, 650500 China

## Abstract

C-reactive protein (CRP) is widely used as a biomarker of inflammation. It plays important roles in innate immunity response as a member of pattern recognition receptors, by binding oxidation-specific epitopes including some intermediates of lipid oxidative chain reaction. The inferred antioxidative ability of CRP was ever demonstrated by only few *in vitro* evidences, and needs to be clarified especially *in vivo*. Herein, we expressed human CRP in three representative non-animal organisms (*Escherichia coli*, *Saccharomyces cerevisiae*, and tobacco) inherently lacking the milieu for CRP signalling, and found CRP did possess an intrinsic antioxidative ability. Heterologous CRP could confer increased oxidative resistance in its recombinant *E*. *coli* and yeast cells and transgenic tobaccos. We also revealed a positive correlation between the antioxidative effect of CRP and its solubility. Only soluble CRP could exhibit distinct antioxidative activity, while the CRP aggregates might be instead toxic (probably pro-oxidative) to cells. Moreover, fusion with hyper-acidic minipeptides could remarkably improve CRP solubility, and meanwhile guarantee or enhance CRP antioxidative ability. These results not only provide a new insight for understanding the etiology of CRP-involved inflammations and diseases, and also endorse a potential of CRP biotechnological applications in developing new pharmaceutical therapies and improving plant oxidative resistance.

## Introduction

C-reactive protein (CRP) is a highly conserved acute-phase protein in human and animals, and tightly associated with numerous inflammations usually elicited by oxidative stress. During inflammation, CRP levels in circulation can sharply increase more than 1000 times within 24 to 72 hours^1^, and it appears almost in all kinds of inflammatory lesions^2–4^. So CRP has become a widely used marker of inflammation. In addition, CRP has been regarded as a non-negligible risk-factor/or mediator for many cardiovascular disorders (CVD)^5,6^, age-related macular degeneration (AMD)^7,8^, and Alzheimer’s disease (AD)^9,10^.

CRP has two conformational isoforms, native pentameric pCRP and modified/monomeric mCRP^11^. pCRP comprises five identical 23 kDa subunits arranged symmetrically around a central pore as a ring^12^. Upon induction by cell membranes/microvesicles or undergoing other transformation mechanisms, pCRP can dissociate into mCRP with an intermediate active isomer of pCRP* or mCRP(m) that exhibit very different functions^13–20^. This might account for the dual but still controversial roles of CRP, *i*.*e*. anti-inflammatory and pro-inflammatory^20–24^, in which mCRP and pCRP* are thought as the major players in the pro-inflammatory actions. In this procedure, the protein becomes more disordered with structural changes that β-sheets decrease meanwhile α-helixes increase^13,19^. mCRP is inclined to aggregation into a matrix-like lattice structure^25–27^ and likely prefers to deposit on the membranes of tissues. For examples, mCRP has been naturally detected with the deposits on blood vessel walls of normal human tissues^28^, and also abundantly found in human atherosclerotic plaques^14^, AMD drusen^7^, and β amyloid plaques in human AD sections^9,10^ probably colocalizing with other aggregated proteins^20^.

CRP can recognize OxLDL and apoptotic cells but not non-oxidized LDL and living cells^29^. In fact, CRP is a kind of pattern recognition receptors (PRRs), belonging to the innate immunity system^30^. Reactive oxygen species (ROS) are unavoidable byproducts of aerobic metabolism. Excessive ROS often produce oxidation-damaged molecules, complexes and cells that must be disposed by macrophages and scavenger cells of the innate immunity system. PRRs mark the oxidation-damaged products accessible to the innate immune system, *i*.*e*. make the dying or damaged cells distinct to the living cells^31^.

By ROS attack, a series of oxidation-specific epitopes (OSEs), *e*.*g*. oxidized phospholipids (OxPLs) and malondialdehyde (MDA)-modified adducts, appeared on apoptotic cell surfaces are recognized by PRRs and consequently involved in the process of apoptotic cell removal^32,33^. Polyunsaturated fatty acyl chains of membrane are preferentially oxidized at the *sn-2* position of glycerol backbone to generate a multitude of truncated OxPLs^34^. Among them, a number of highly reactive OxPL derivatives contain groups such as aldehydes or carboxylic acids at the end of their *sn-2* position. These polar moieties might no longer stay within the low dielectric hydrocarbon phase and tend to extrude into the adjacent aqueous phase^35^. OxPLs preferentially assemble together and form ‘patches’ that act as nanosensors to be recognized by PRRs of the innate immune system^31,33,36^.

Among OxPLs, oxidized phosphatidylcholine (OxPC) is the specific pattern recognition ligand of CRP^37^. Actually, CRP binds OxLDL and apoptotic cells by recognizing their common cognate epitope, OxPC^29^. Each CRP subunit has a binding site for OxPC^12,29^. Phosphatidylcholine (PC) is the major lipid component on membranes and lipoproteins, and most frequently susceptible to oxidative conversion into OxPCs under ROS attack. CRP can bind a variety of OxPC species, including high-reactive PC-peroxiradicals and PC-hydroperoxides that are the intermediates of lipid oxidative chain reaction^38^. This action may shield reactive OxPCs from interacting with unoxidized phospholipids, thus block the progression of oxidative chain reactions and protect cells from oxidative damages, implying that CRP is likely of an intrinsic antioxidative ability.

Nevertheless, there are very few reports about the direct antioxidative activity of CRP. Only two *in vitro* studies showed that CRP could inhibit oxidation of LDL and phospholipid liposomes at physiological concentrations by using its recognition properties^39,40^. Usually, the recognition function is thought as the requisite for CRP-mediated complement activation, leading to the pro-inflammatory role of CRP as well as its opposite activities (*e*.*g*. anti-pneumococcal, anti-atherosclerotic, anti-arthritic) mainly observed in animal models of inflammatory diseases^22^. However, these protective effects none are derived from the experimental animals deficient in CRP-signalling pathway thus can not absolutely point to CRP alone. Therefore, the puzzle whether or not CRP has an intrinsic antioxidative role remains to be clarified, especially needing *in vivo* evidences. Herein, we deliberately attempted to verify the antioxidative role of heterologous CRP in several non-animal organisms such as *E*. *coli*, yeast and higher plants interiorly devoid of the milieu for CRP signalling. We unraveled that CRP did own an inherent antioxidative ability, the actual effect of which *in vivo* was highly determined by its solubility.

## Results

### Heterologous CRP confers increased oxidative resistance in *E*. *coli*

**Figure 1:**
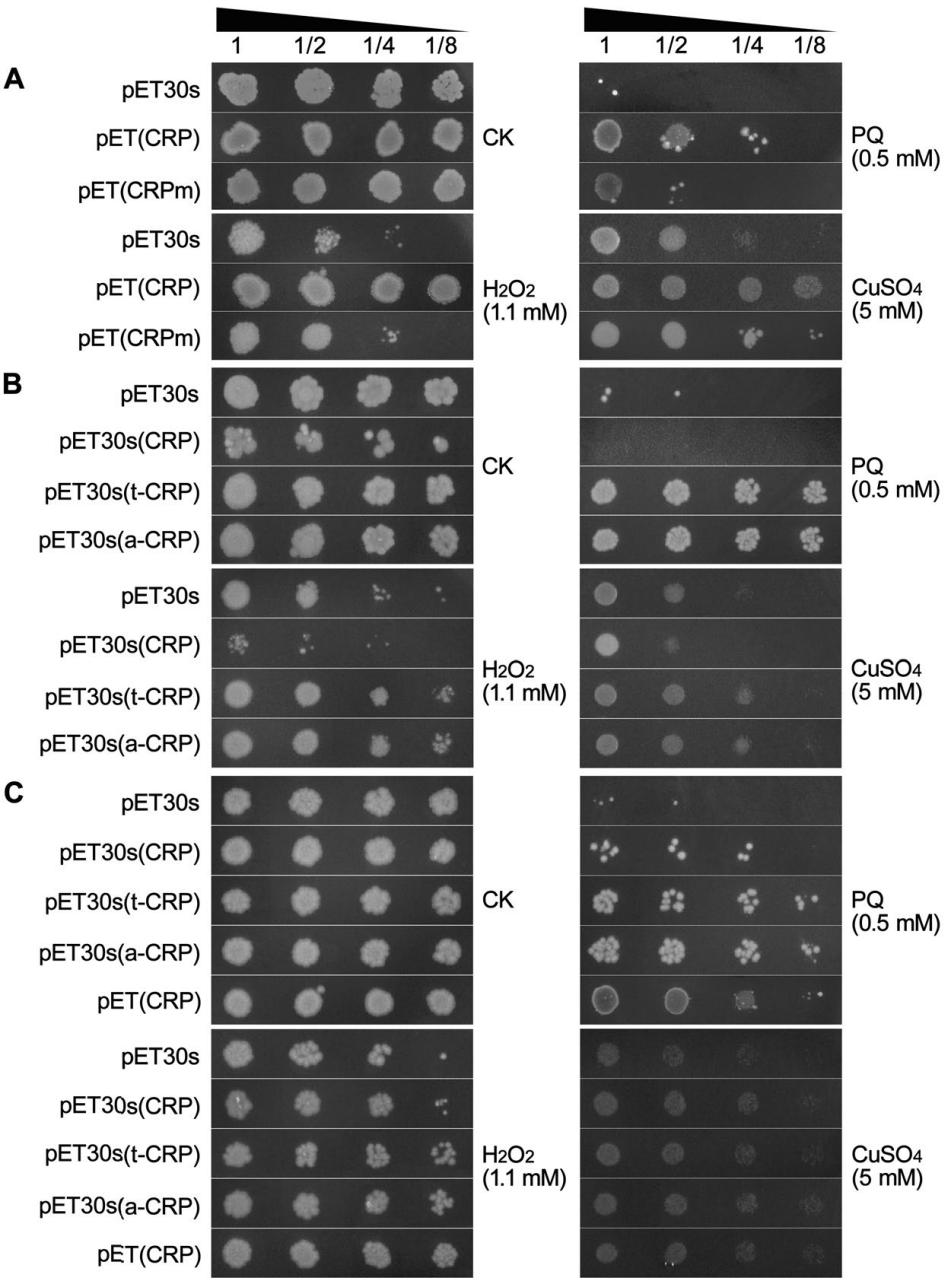
Dot-plating test with serial dilutions (1-, 2-, 4-, 8- fold) to compare the colony growth of *E*. *coli* recombinant strains of pET(CRP), pET(CRPm), pET30s(CRP), pET30s(t-CRP), pET30s(a-CRP) and the control strain of pET30s, under normal condition (CK) and diverse oxidative stresses of 1.1 mM H_2_O_2_, 0.5 mM PQ, and 5 mM CuSO_4_. (**A**,**B**) *E*. *coli* cells with pre-induction. (**C**) *E*. *coli* cells without pre-induction.

Firstly, we amplified the coding fragment of the mature chain of human CRP, and constructed its prokaryotic expression vector pET(CRP) and the derived pET(CRPm) containing a mutant of CRP (F66Y/E81K) unable to bind phosphocholine. Then we introduced them into *E*. *coli* BL21(DE3) strain to evaluate their antioxidative ability under three commonly used oxidative stresses of H_2_O_2_, paraquat (PQ) and CuSO_4_, using dot-plating tests for *E*. *coli* cells with pre-induction. As shown in Fig. 1A, the cell growth of all tested *E*. *coli* strains were similar under normal conditions (CK), but inhibited differentially under various oxidative stresses (1.1 mM H_2_O_2_, 0.5 mM PQ, and 5 mM CuSO_4_). Therein, the colony status of pET(CRP)-recombinant strain was remarkably better than the control strain of pET30s. This was further confirmed by dynamic curve assays on *E*. *coli* growth under oxidative stresses of 0.5 mM PQ and 5 mM CuSO_4_ (Supplementary Fig. 1). However, the recombinant *E*. *coli* strain of pET(CRPm) showed a much worsen colony status as compared to that of pET(CRP), even resembling the control strain (Fig. 1A). These results implied that the heterologous CRP mediated an increased antioxidative ability in *E*. *coli* making cells more resistant to oxidative stresses, and this effect was crucially correlated with its binding site specific to oxidized phosphocholine.

### The antioxidative activity of CRP largely depends on its solubility

**Figure 2:**
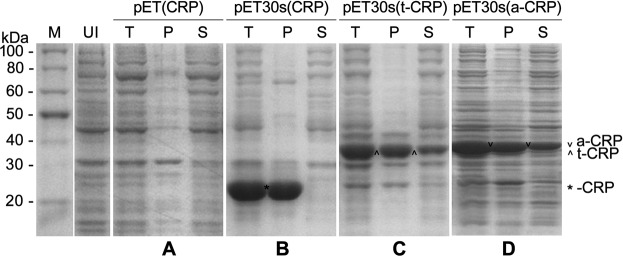
Protein expression in *E*. *coli* of various CRP recombinant vectors including pET(CRP) (**A**), pET30s(CRP) (**B**), pET30s(t-CRP) (**C**) and pET30s(a-CRP) (**D**), analyzed by SDS-PAGE. M: protein molecular weight marker; UI: crude lysate of uninduced bacterial cells; T: crude lysate of induced bacterial cells; P, S: the pellet and supernatant fractions of induced bacterial cell lysate (T) after centrifugation, respectively. Full-length gels are presented in Supplementary Fig. 9.

We further analyzed the expression of CRP in pET(CRP)-recombinant *E*. *coli* strain, and surprisingly failed to detect the protein band of CRP on the SDS-PAGE gel (Fig. 2A). Such low level of CRP expression might be attributed to the introduction of a new ribosomal binding site (RBS) in pET(CRP) to substitute the T7 g10 leader sequence of high translation efficiency in the original pET-serial vectors. Therefore, we reconstructed the expression vector pET30s(CRP) by inserting the *CRP* gene behind the *Nco* I site in the backbone of pET30s. In this case, CRP expression was drastically enhanced to an extreme level, but the recombinant CRP (*i*.*e*. -CRP) was almost insoluble and aggregated into inclusion bodies (Fig. 2B). Consequently, we exploited the fusion strategy to improve the soluble expression of CRP, by using two known *cis*-acting solubility-enhancers, *i*.*e*. hyper-acidic fusion minipeptides, TUA2 (41 aa) and ATS (43 aa). We added TUA2 or ATS moiety at the N-terminal of CRP to create the fusion expression vectors pET30s(t-CRP) and pET30s(a-CRP), respectively. The expressed fusion proteins TUA2-CRP (*i*.*e*. t-CRP) and ATS-CRP (*i*.*e*. a-CRP) were commonly of high level (Fig. 2C,D) and significant increase in solubility (37.5% and 38.9%, respectively) (Supplementary Fig. 2), indicating both hyper-acidic fusion minipeptides are substantially effectual to enhance CRP solubility.

Then, we used dot-plating tests again to investigate the antioxidative ability of newly constructed CRP-recombinant *E*. *coli* strains with pre-induction. Under normal condition (CK), the recombinant strains harboring either CRP fusion expression vectors (pET30s(t-CRP), pET30s(a-CRP)) had normal colony status similar to the control strain of pET30s, while the cell growth of pET30s(CRP)-recombinant strain was unexpectedly suppressed. In contrast, all these strains were differentially inhibited for growth under diverse oxidative stresses (1.1 mM H_2_O_2_, 0.5 mM PQ, and 5 mM CuSO_4_). Therein, the recombinant strains expressing TUA2/ATS-CRP (*i*.*e*. t/a-CRP) fusions were less suffered and exhibited a much better colony status, whereas the pET30s(CRP)-recombinant strain showed more severe suppression, as compared to the control strain (Fig. 1B).

Based on above results, we proposed that the antioxidative activity of CRP was likely dependent on its solubility to a large extent. CRP is soluble in case of low expression level (*e*.*g*. in pET(CRP)-recombinant strain), and thus exhibits its intrinsic antioxidative role. Overexpression of CRP (*e*.*g*. in pET30s(CRP)-recombinant strain) causes the formation of aggregates into the inclusion bodies. Such folding-aberrant CRP is probably inactive and instead toxic to *E*. *coli* growth. When fusing with the solubility-enhancers, high amount of CRP protein fusions (TUA2/ATS-CRP) (*e*.*g*. in pET30s(t/a-CRP)-recombinant strains) are still of considerable solubility, and retain the antioxidative activity to protect *E*. *coli* cells from oxidative impairments.

To substantiate this hypothesis, we conducted additional dot-plating tests for all forms of CRP recombinant strains, using *E*. *coli* cells without pre-induction. Conceivably, the expression of recombinant proteins only induced on solid plates is quite slow, thus of a relatively low level but high solubility. In these cases, as judged by comparing the colony growth status, low amount of CRP on longer suppressed the growth of pET30s(CRP)-recombinant *E*. *coli* strain, and instead rehabilitated its antioxidative role under diverse oxidative stresses. Certainly, the recombinant strains expressing more soluble TUA2/ATS-CRP fusions even showed a relatively stronger antioxidative ability, and pET(CRP)-recombinant strain natively of low CRP expression still maintained a robust antioxidative activity (Fig. 1C). Obviously, these results are well in agreement with our presumption that the antioxidative ability of CRP is highly commensurate to its protein solubility.

### Heterologous CRP confers increased oxidative resistance in *S*. *cerevisiae*

**Figure 3:**
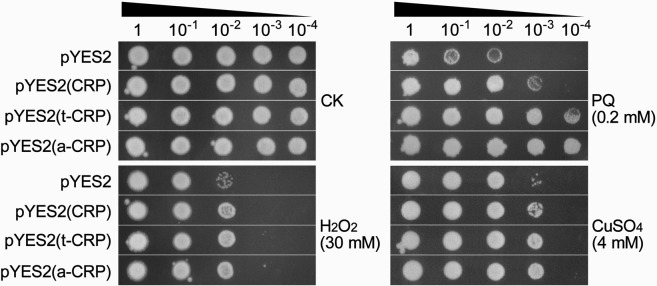
Dot-plating test with serial dilutions (1-, 10-, 100-, 1000-, 10000- fold) to compare the colony growth of yeast cells with pre-induction among the recombinant strains of pYES2(CRP), pYES2(t-CRP), pYES2(a-CRP) and the control strain of pYES2 under normal condition (CK) and different oxidative stresses of 30 mM H_2_O_2_, 0.2 mM PQ, and 4 mM CuSO_4_.

We further evaluated the antioxidative function of CRP in single eukaryotic cell such as *S*. *cerevisiae*, using yeast dot-plating tests with serial dilutions. Generally, as compared to that in *E*. *coli* system, expression of recombinant proteins in *S*. *cerevisiae* is hardly efficient. Thus, even after a period of pre-induction for yeast cells, the expressed CRP is still likely below a threshold level triggering severe aggregation, and retains a certain degree of solubility. Meanwhile, the expressed TUA2/ATS-CRP fusions are theoretically more soluble. We found that all tested yeast strains showed differential growth inhibition under diverse oxidative stresses (30 mM H_2_O_2_, 0.2 mM PQ, and 4 mM CuSO_4_), despite of a similar normal colony status under unstressed condition (CK). As expected, pYES2(CRP)-recombinant yeast strain had a notably better colony growth than the control strain of pYES2 plasmid, which was yet outperformed by both recombinant strains carrying CRP fusion yeast expression vectors, pYES2(t-CRP) and pYES2(a-CRP) (Fig. 3). These observations indicated that the heterologous CRP enhanced the antioxidative ability of *S*. *cerevisiae*. Additionally, this effect could be fortified by fusing with hyper-acidic partners TUA2, ATS for a higher solubility, again reflecting a positive correlation between CRP-mediated antioxidative activity and its solubility. Overall, these results in yeast were in line with above findings in bacteria.

We also performed dot-plating tests using non-induced yeast cells, and found no difference in the colony growth status among the control and any CRP recombinant yeast strains, under both normal and stress conditions (Supplementary Fig. 3). This can be explained by an extremely low expression of CRP and its fusion forms (t/a-CRP) in *S*. *cerevisiae* only induced on plates, probably being with a negligible activity.

### Heterologous CRP confers increased oxidative resistance in tobacco

The antioxidative ability of CRP was well verified in *E*. *coli* and yeast, so we envisioned that CRP could also confer a similar role in higher plants. Regarding this, we constructed plant expression vectors of CRP and its fusion forms (TUA2-CRP, ATS-CRP), and performed a serial of antioxidation analyses on their transgenic tobacco lines (briefly termed CRP, t-CRP, a-CRP, respectively).

**Figure 4:**
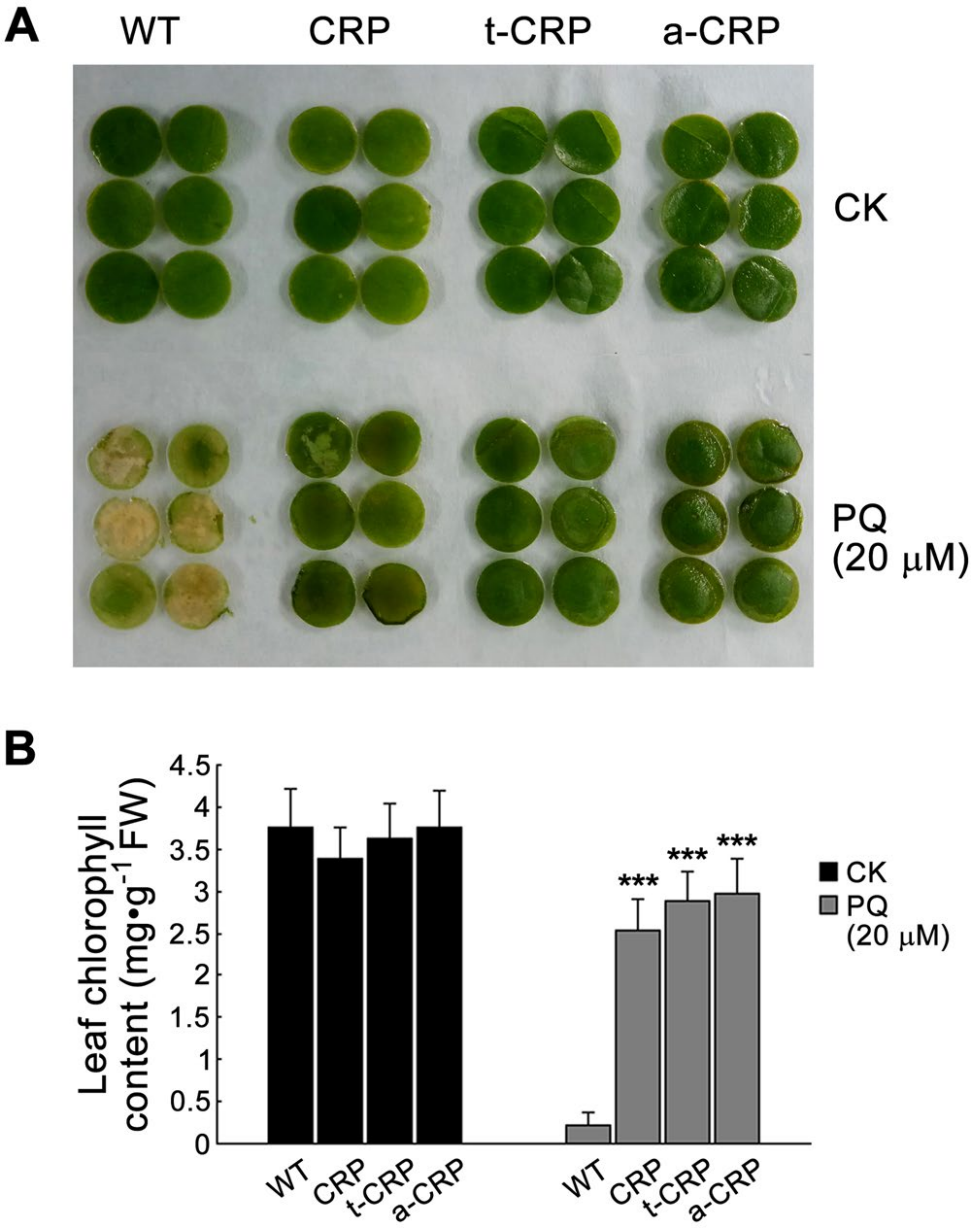
Preliminary antioxidation analysis of various CRP transgenic tobaccos of T_0_ generation. For a chlorosis test (**A**), leaf discs of 4-week-old WT and transgenic tobaccos (CRP, t-CRP, a-CRP) growing in greenhouse were submerged into deionized water (CK) or 20 µM PQ solution for 7 d in a growth chamber. After photo-recording, their chlorophyll contents were measured (**B**). Statistical significance was determined by Student’s t test (n = 3, two replicates per experiment, data are the means ± SD, ***P < 0.001).

Considering that oxidative stress can impair photosynthesis apparatus, we initially conducted leaf chlorosis test to evaluate the oxidative resistance of various CRP transgenic tobaccos of T_0_ generation. After a consecutive immersion for 7 d, wild-type (WT) tobacco exhibited more severe chlorosis on leaf discs than various CRP transgenic tobaccos in PQ (20 μM) solution, while the control set of leaf discs (CK) similarly stayed green in deionized water (Fig. 4A). Upon PQ treatment, the residual chlorophyll content in leaf discs of WT tobacco was only 5.8% of its control, whereas CRP, t-CRP and a-CRP transgenic tobaccos still retained 74.6%, 79.6% and 79.2% chlorophyll in turn, extremely higher than that in WT tobacco (Fig. 4B). These results preliminarily revealed that both CRP and its fusion forms could introduce an effectual antioxidative activity in tobacco, protecting leaves from chlorophyll destruction by oxidative damages.

**Figure 5:**
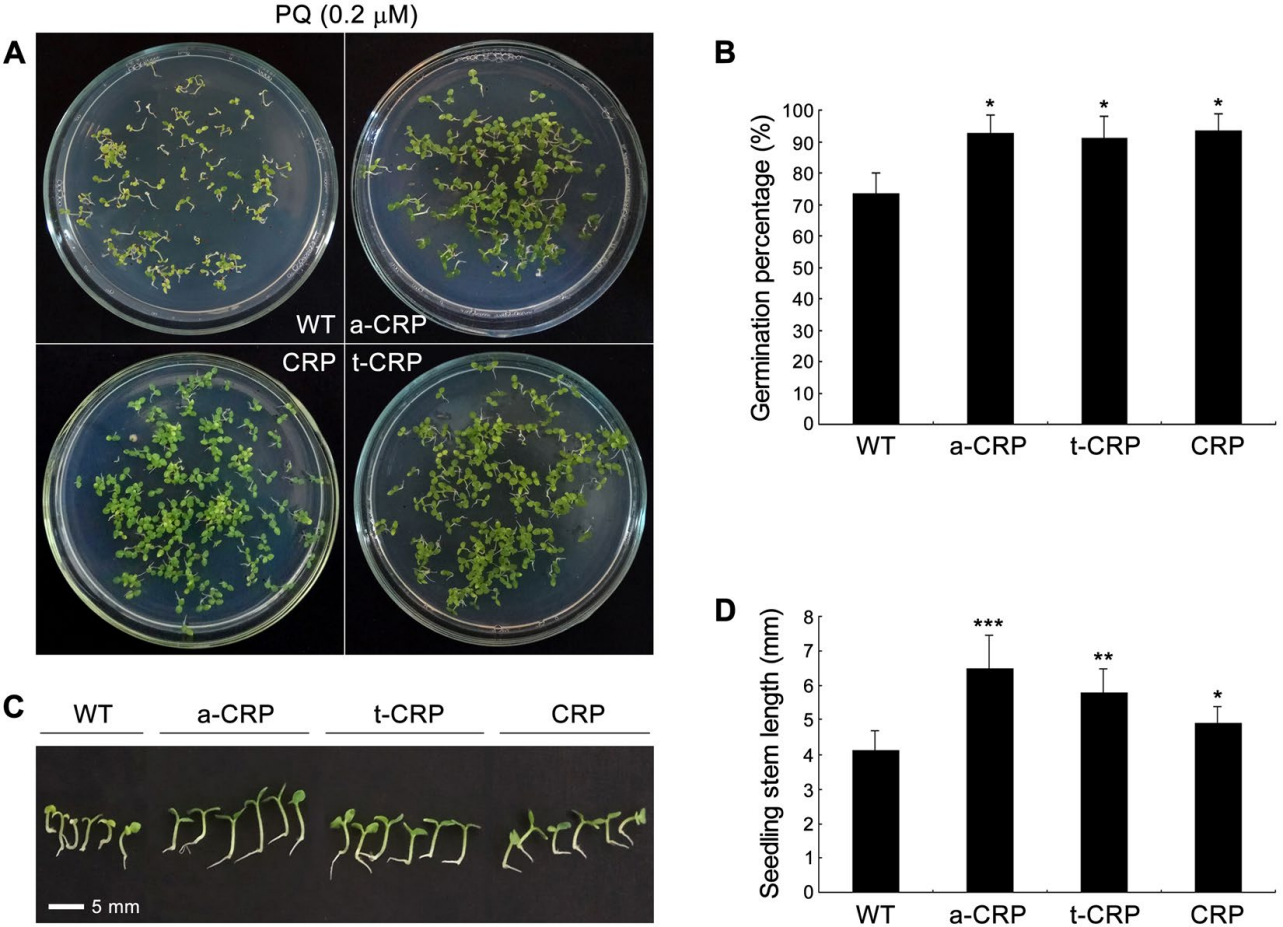
Seed germination test of various CRP transgenic tobaccos under moderate oxidative stress. Sterile seeds of WT and transgenic tobaccos (a-CRP, t-CRP, CRP) were germinated in a growth chamber on MS medium containing 0.2 μM PQ for 10 d, and then assessed for the germination profile (**A**), germination rate (**B**), seedling status (**C**), and stem-length of seedlings (**D**). Statistical significance was determined by Student’s t test (n = 3, >80 seeds per replicate experiment, data are the means ± SD, *P < 0.05, **P < 0.01, ***P < 0.001).

Then we examined the influence of oxidative stress on seed germination of various CRP transgenic tobacco lines. After sowing on Murashige & Skoog (MS)-agar plates for 10 d, the sterile seeds of WT and all transgenic tobaccos showed differential germination with reduced rates in the medium containing the oxidation inducer of PQ (Fig. 5A; Supplementary Fig. 4A). Therein, under an oxidative stress elicited by 0.2 μM PQ, the seed germination rates of WT and a-CRP, t-CRP, CRP transgenic tobaccos were 73.5%, 92.3%, 91.1% and 93.7%, respectively (Fig. 5B). Moreover, upon a severe stress of 20 μM PQ, the germination percentage of WT tobacco seeds declined gravely to 49.4%, while a-CRP, t-CRP and CRP transgenic tobaccos still maintained a relatively higher seed germination rate of 81.3%, 83.9% and 82.5%, respectively (Supplementary Fig. 4B). These data indicated all forms of CRP transgenic tobaccos commonly possessed a better seed germination than WT tobacco under both oxidative stresses. In addition, WT seedlings exhibited remarkable chlorosis and short stems. Contrastively, the seedlings of various CRP transgenic tobaccos considerably remained green and had notably longer stems (Fig. 5C; Supplementary Fig. 4C). Intriguingly, both a-CRP, t-CRP transgenic tobaccos overall had seedlings of significantly higher stem-length than CRP transgenic tobacco (Fig. 5C,D; Supplementary Fig. 4C). Taken together, these findings implied that the oxidative stress could severely inhibit seed germination and seedling growth in tobacco, and this suppression could be alleviated by CRP, likely more effectually by more soluble ATS-CRP, TUA2-CRP fusions. Apparently, these heterologous proteins could provide tobacco an increased antioxidative ability, thus protecting seed vigor and seedling growth from oxidative deterioration.

**Figure 6:**
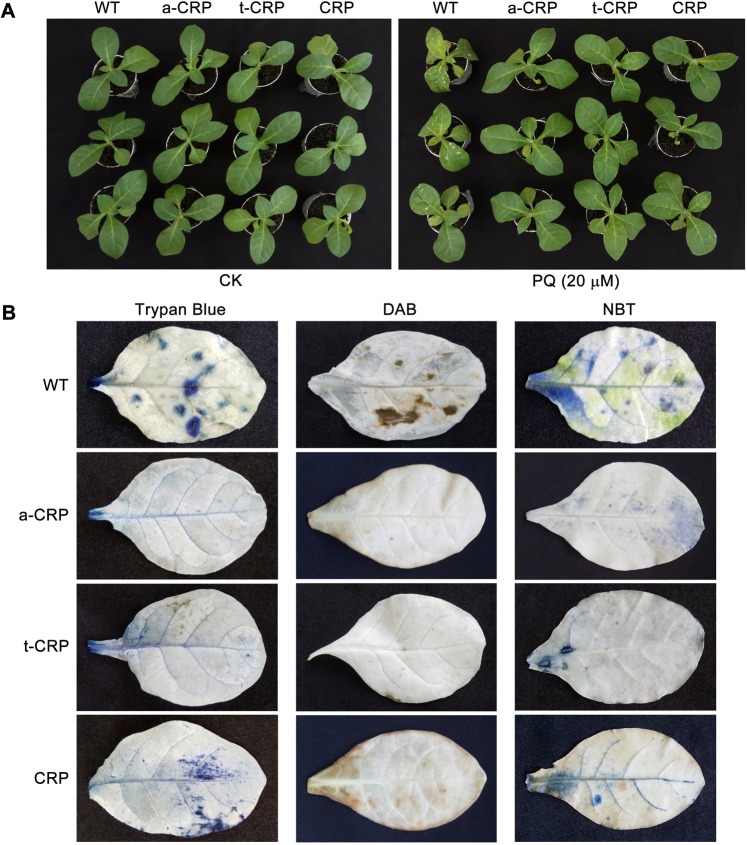
Leaf spray test and histochemical staining of various CRP transgenic tobaccos with a oxidative treatment. (**A**) Small plants (1-week growth in greenhouse after 1-month sterile cultivation of seedlings) of WT and transgenic tobaccos (a-CRP, t-CRP, CRP) were daily leaf-sprayed with deionized water (CK) or 20 µM PQ solution for 7 d, then the injury spots on leaves were assessed. (**B**) Intact leaves (daily sprayed with 20 µM PQ for 7 d) of WT and transgenic tobaccos (a-CRP, t-CRP, CRP) were detected for dead cells by Trypan Blue staining (indigo mark), for H_2_O_2_ by DAB staining (dark brown mark), and for O_2_^−^ by NBT staining (blue mark), respectively.

We further conducted PQ spray tests to investigate the antioxidative ability of various CRP transgenic tobacco plants of T_1_ generation. After daily leaf spray for 7 d, WT and all transgenic tobaccos identically had normal growth profiles when sprayed with deionized water (CK), but showed different degrees of oxidative injury on leaves sprayed with PQ (20 μM) solution. Therein, lots of injury spots could be seen on WT tobacco, but only a little on CRP transgenic tobacco. Contrastly, no evident lesions were detected on a-CRP and t-CRP transgenic tobaccos (Fig. 6A). Moreover, a similar scenario appeared when spraying with PQ (30 μM) solution for 3 d (Supplementary Fig. 5). Subsequently, these sprayed leaves were elaborately analyzed as the following.

Since oxidation stress can induce ROS burst leading to cell apoptosis, the resultant lesions should accumulate excessive ROS and lots of dead cells that can be directly visualized by *in situ* histochemical staining. Trypan Blue is routinely used to monitor membrane integrity and cell viability by marking the dead cells, while 3,3′-diaminobenzidine (DAB) and nitroblue tetrazolium (NBT) are generally utilized to detect the accumulation and location of two types of ROS species, H_2_O_2_ and superoxide radical (O_2_^−^), respectively. Unsurprisingly, we found that WT and all CRP transgenic tobaccos showed differential staining signs on leaves sprayed with PQ (20 μM) solution. Therein, by Trypan Blue staining (indigo mark), many unique staining aggregates appeared on WT tobacco, while only diffusive spots on CRP transgenic tobacco. In contrast, no visible staining signs were detected on a-CRP and t-CRP transgenic tobaccos. Meanwhile, similar staining profiles were obtained when using other two methods, *i*.*e*. DAB staining (dark brown mark) and NBT staining (blue mark) (Fig. 6B). These observations intuitively demonstrated that upon oxidative threats, dead cells and ROS accumulated less in CRP transgenic tobacco, and rarely in both a-CRP and t-CRP transgenic tobaccos, as compared to those in WT tobacco.

**Figure 7:**
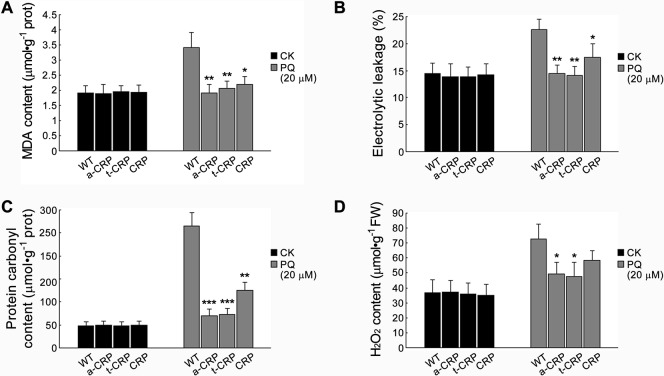
Oxidation-associated physiological changes in various CRP transgenic tobaccos after oxidative treatment. Leaves (daily sprayed with 20 µM PQ or deionized water (CK) for 7 d) of WT and transgenic tobaccos (a-CRP, t-CRP, CRP) were measured for MDA content (**A**), electrolyte leakage (**B**), protein carbonyl content (**C**), and H_2_O_2_ content (**D**). Statistical significance was determined by Student’s t test (n = 3, two replicates per experiment, data are the means ± SD, *P < 0.05, **P < 0.01, ***P < 0.001).

Additionally, we examined the physiological changes in all forms of CRP transgenic tobaccos upon 7-d daily treatment with 20 μM PQ, and hence measured several main oxidation-associated physiological parameters. Therein, the contents of MDA and protein carbonyl as well as the electrolyte leakage, as routinely used oxidation hallmarks, are generally parallel to the degrees of oxidative damages, and the content of H_2_O_2_ is an important indicator of cellular ROS level. Overall, we found that these physiological indices each were almost identical among WT tobacco and any transgenic lines without stress treatments (CK), but notably lower in CRP transgenic tobacco and lowest in both a-CRP and t-CRP transgenic tobaccos under PQ stress when comparing those in WT tobacco (Fig. 7). In detail, upon PQ oxidative treatments, the content of MDA and the electrolyte leakage individually increased up to 1.8-, 1.6- fold in WT tobacco and nearly 1.3-, 1.2- fold in CRP transgenic tobacco, but almost kept unchanged in both a-CRP and t-CRP transgenic tobaccos (Fig. 7A,B). Meanwhile, the contents of protein carbonyl and H_2_O_2_ separately raised up to 5.5-, 2- fold in WT tobacco and about 2.5-, 1.7- fold in CRP transgenic tobacco, yet only approximately 1.5-, 1.3- fold in both a-CRP and t-CRP transgenic tobaccos (Fig. 7C,D). These physiological analyses quantitatively showed that upon oxidative stress, CRP transgenic tobacco had lower amount of ROS accumulation and suffered with less degrees of oxidative damages on membrane lipids and cellular proteins as compared to WT tobacco, and this differential was more evident in both a-CRP and t-CRP transgenic tobaccos.

Moreover, we also compared PQ (20 µM)-induced changes in the activities of three cellular antioxidases, catalase (CAT) (Supplementary Fig. 6A), peroxidase (POD) (Supplementary Fig. 6B) and superoxide dismutase (SOD) (Supplementary Fig. 6C). Overall, under PQ stress, WT and various CRP transgenic tobaccos together showed a significant increase in the activities of CAT and POD, but seemingly with an invariant SOD value. Nevertheless, no remarkable difference in selected enzymatic activities was found among WT and any CRP transgenic tobaccos, whatever with or without oxidative treatments. These data substantially indicated that the predominant antioxidative ability in transgenic tobaccos was in deed conferred by heterologous CRP or its fusions and not likely by cellular antioxidases.

Conclusively, all these results iterated that the heterologous CRP authentically exerted an effectual antioxidative activity in tobacco thus reducing ROS accumulation, damages on membranes and macromolecules, cell apoptosis and tissue injuries under oxidative stress, and this effect could be further surpassed by its hyper-acidified fusion forms (ATS-CRP, TUA2-CRP) with higher solubility.

**Figure 8:**
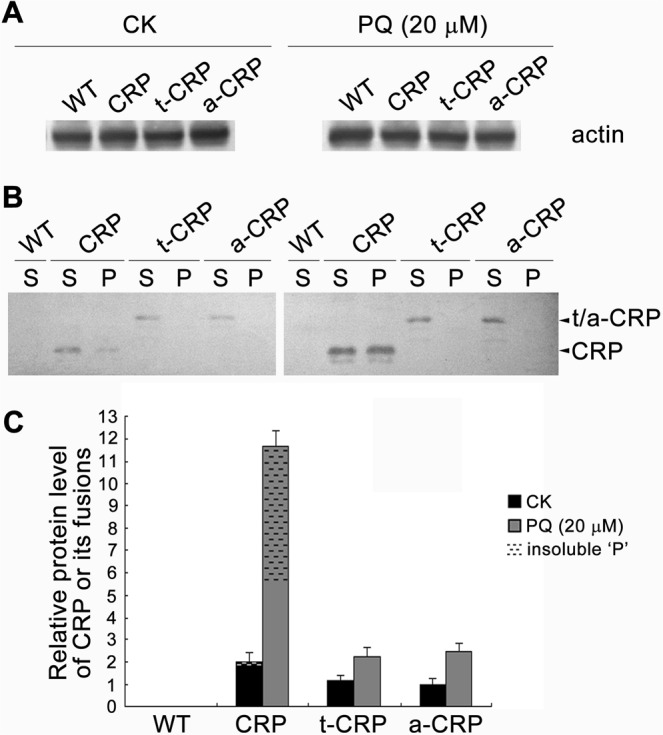
Changes in the expression of CRP and its fusions at protein level in their transgenic tobaccos upon oxidative treatment. Leaves (daily sprayed with 20 µM PQ or deionized water (CK) for 7 d) of WT and transgenic tobaccos (CRP, t-CRP, a-CRP) were subjected for protein extraction plus fractionation into ‘S’, ‘P’ samples and subsequent immunoblotting with CRP polyclonal antibody. Actin (‘S’ fraction) was used as the internal reference (**A**) to calibrate the total expression (‘S’ + ‘P’) of CRP protein or its fusions t/a-CRP (**B**) in various transgenic tobaccos by membrane-band grey densitometric estimation. The lowest ratio was standardized as 1 to obtain the relative protein levels of CRP and its fusions for comparison (**C**). Data represent the means ± SD for three separate experiments. Full-length blots are presented in Supplementary Fig. 10.

Finally, we checked the expression of CRP and its fusion forms in their transgenic tobaccos. Semi-quantitative reverse transcriptional PCR (RT-PCR) showed that CRP (or its fusions) expression at mRNA level were quite similar in all transgenic tobaccos without stress treatments (CK), while upregulated significantly and differentially under PQ (20 µM)-elicited oxidative stress, *i*.*e*. about 5-fold in CRP transgenic tobacco, but 2-fold in both t-CRP and a-CRP transgenic tobaccos (Supplementary Fig. 7). We further used immunoblotting to detect various CRP expressions at protein level, and found that the total amount of expressed CRP was about 1.7-, 2- fold in turn as that of its fusion form t-CRP, a-CRP in their corresponding transgenic tobaccos with the control treatments (CK). Contrastively, upon PQ stress, expression of these heterologous proteins increased remarkably with different degrees, *i*.*e*. nearly 6-, 2-, 2.5- fold in CRP, t-CRP and a-CRP transgenic tobaccos, respectively. Interestingly, when comparing the supernatant (S) and insoluble fraction (P) of each recombinant protein expressed in transgenic tobaccos, only CRP was observed with aggregates of trace amount under CK condition but an astonished portion (up to 52%) upon PQ treatment (Fig. 8). These data showed that the oxidative induction could significantly boost the expression of CRP and its fusion forms in their transgenic tobaccos. With the increase in protein amounts, expressed CRP was prone to formation of aggregates and thus exhibited an attenuated antioxidative ability, while its hyper-acidified fusions (TUA2-CRP, ATS-CRP) still maintained a very high solubility and hence exerted a stable or even enhanced antioxidative activity. This might be the right interpretation for aforementioned antioxidative predominance in transgenic tobaccos expressing TUA2/ATS-CRP fusions versus that of CRP, and again highlighted that the antioxidative activity of CRP was highly and positively correlated with its protein solubility.

## Discussion

In human and animals, inflammation is usually elicited by excessive ROS under pathological oxidative stress, and to a large extent it can be viewed parallel to oxidation. CRP is tightly involved in inflammation and thus certainly associated with oxidation. So far both pro-inflammatory and anti-inflammatory roles of CRP have been reported but still under debates^14,15,17,19,22^, the exact relationship of CRP with inflammation remains elusive. This confusion might be due to a fact that the current studies almost put central attention on the signalling role of CRP in the innate immunity and overlooked its direct responses to oxidation. Noting that CRP, as a PRR member, can bind oxidation-specific epitope, OxPC in the innate immunity^29,31^, a putative antioxidative role was ever inferred from its recognition properties but only scarcely evaluated *in vitro*^39,40^. In this study, we expressed CRP heterologously in non-animal organisms thus precluding its signalling role, and presented for the first time the *in vivo* concrete evidences for this deduction.

Overall, we found that CRP did possess an intrinsic antioxidative ability and confer an enhanced oxidative resistance in their recombinant *E*. *coli* and yeast cells and transgenic tobaccos. We also revealed that this effect of CRP was highly dependent on its solubility. Generally, the solubility is considerably determined by the amount in most proteins including aggregation-prone CRP^41,42^. Herein, in *E*. *coli*, pET(CRP)-derived recombinant CRP was of poor expression seemingly below the aggregation threshold in whatever occasions with or without pre-induction (Fig. 2A), and hence constantly had a very high solubility, resulting in a stable and potent antioxidative activity to protect bacterial cell growth against diverse oxidative stresses (Fig. 1A,C). Contrastively, pET30s(CRP)-derived CRP could express dramatically upon IPTG induction but almost aggregated into inclusion bodies (Fig. 2B). Such folding-abnormal CRP aggregates were likely inactive and rather become pro-oxidative to inhibit *E*. *coli* growth (Fig. 1B). However, this CRP of high expression potential restored its antioxidative ability when turning down its expression level in case of no pre-induction (Fig. 1C), probably due to an increased solubility. Most noticeably, both CRP fusions (TUA2/ATS-CRP) derived from the refined pET30s(t/a-CRP) vectors always showed a distinct antioxidative effect likely irrespective of the expression level (Fig. 1B,C). This might be ascribed to their relatively high solubility that was securely improved by fusing with hyper-acidic minipeptides (TUA2, ATS) (Fig. 2C,D**)**. In *S*. *cerevisiae* with relatively low capacity for recombinant protein expression, the obtained results were overall in line with the findings in *E*. *coli*, especially resembling the observations in bacterial cells without pre-induction. Both hyper-acidified CRP fusions outperformed CRP to endue their recombinant yeast cells with a much better colony growth under diverse oxidative stresses (Figs 1C and 5). Likewise, both CRP fusions surpassed CRP to exert a more predominant antioxidative activity in their transgenic tobaccos, by reducing ROS accumulation, chlorophyll destruction, damages on membranes and proteins, cell apoptosis and tissue injuries meanwhile protecting seed vigor and seedling growth under PQ-elicited oxidative stress, as demonstrated by our detailed analyses (Figs 4–7; Supplementary Figs 4 and 5).

Moreover, we could also perceive a dynamic competition between the expression level and protein solubility to affect the antioxidative activity of CRP in *E*. *coli*. Above the threshold level of CRP aggregation, even a weak expression, *e*.*g*. in pET30s(CRP) strain without pre-induction, may still not absolutely generate the soluble/active CRP, and at least a certain amount of which can form toxic aggregates, thus somewhat offsetting the antioxidative effect of the soluble portion to give bacterial cells a compromised oxidative resistance even much weaker than that in pET(CRP)-recombinant strain (Fig. 1C). With the increase in expression, more CRP aggregates are produced with more severe compromise in functionality, and this tendency ultimately reaches the scenario as seen in pET30s(CRP) strain with pre-induction (Fig. 1B). Nevertheless, this competition is likely very mild on both occasions of expression (*i*.*e*. with or without pre-induction) in pET30s(t/a-CRP) strains because of expressing more soluble CRP fusion forms, thus bestowing *E*. *coli* cells a similar eminent antioxidative profile as found in pET(CRP)-recombinant strain (Fig. 1B,C). Probably, this mechanistic perception could also be applicable to explain the antioxidative difference in recombinant yeast strains and transgenic tobaccos expressing CRP or its fusions. Especially keeping an eye on transgenic tobaccos, upon PQ oxidative stress, CRP protein totally expressed about 5 times more than its fusion forms that are almost fully soluble. However, highly expressed CRP was inclined to formation of aggregates with a final portion up to 52%, although its soluble fraction exceeded the double amount of each CRP fusion (Fig. 8). The aggregates-mediated pro-oxidative toxicity at least partially offset the antioxidative effect of the soluble portion, hence leading to an attenuated oxidative resistance in CRP transgenic tobacco which should be comparable to that in transgenic tobaccos expressing the soluble CRP fusions.

Taken together, it can be concluded that hyper-acidic fusion minipeptides not only convey an increased solubility to aggregation-prone CRP, but also escort its intrinsic antioxidative ability no matter what expression level. Additionally, regarding another notion that it seems difficult to modulate individual expression of heterologous proteins especially in higher organisms, thereby it is recommended of the least risk to preferentially choose hyper-acidified CRP fusions rather than the native CRP when engineering oxidative resistance with a guaranteed enhancement. Particularly, this would be of great importance for those staple economic plants with a compelling need to cope with oxidative threats induced by numerous environmental challenges^43,44^.

Believably, CRP achieves its antioxidative effect by taking advantages of its membership of PRR that can specifically bind OxPCs including high-reactive species^31,38^. This recognition function of CRP may prevent reactive OxPCs from interacting with unoxidized phospholipids, thus block the progression of oxidative chain reactions on membranes, and reduce ROS accumulation and oxidative damages. It seems like the protective mechanism of lipocalins against oxidative stress in animals and plants by binding the ligands of lipid oxidation products to prevent peroxidation^45,46^. In fact, this notion was considerably substantiated in our antioxidation analyses on various CRP transgenic tobaccos and the recombinant *E*. *coli* strain of a CRP mutant. Therein, as compared to WT tobacco, less ROS (*e*.*g*. H_2_O_2_, O_2_^−^) accumulated in all kinds of CRP transgenic tobaccos with less oxidative damages (on membranes, proteins, chlorophyll, and even cells) or weaker inhibition for growth under PQ stress (Figs 4–7; Supplementary Figs 4 and 5). In *E*. *coli*, the antioxidative role of recombinant CRP was severely inactivated when the major binding loci specific to OxPCs^12,47^ was eliminated by mutagenesis, while the remnant effect that weakly protected *E*. *coli* from oxidative stress (Fig. 1A) might be ascribed to other available minor binding sites^48,49^. Moreover, this viewpoint can also be suitable to explain the duplicitous roles of CRP on oxidation (*i*.*e*. antioxidative and pro-oxidative) that are highly determined by its solubility. Only the soluble forms of CRP have exposed binding sites for OxPCs thus exhibiting distinct antioxidative activity, whereas CRP aggregates make these sites embedded and instead become pro-oxidative and toxic to cells. Noteworthily, protein aggregates-mediated pro-oxidation has already been reported previously and defined as a pathogenic factor^50^. For instance, aggregation of amyloid β42 (Aβ42) peptide can induce oxidative stress during the pathogenesis and progression of AD^51,52^.

In addition, noting that pCRP, pCRP*/mCRPm, mCRP and its reduced form all can bind PC moiety-contained components^13,16,19,53^, it appears that this binding action does not absolutely require a complete native folding for CRP, at least in which the intra-subunit disulfide bond can be dispensable. In this study, the soluble recombinant CRP and its hyper-acidified fusions expressed in the reductive cytoplasm of *E*. *coli*, yeast and tobacco might not be able to ultimately fold into the native form as pCRP, but could be rationally proposed with similar tertiary structures as reduced mCRP or its analogues of folding intermediates. According to a stepwise folding pathway of CRP reported very recently^54^, they should have already been structured to a conformation of near-native core by folding spontaneity at the first stage, which is likely sufficient for correct exposures of the binding sites to OxPC and thus executing the antioxidative activity. In this point, the subsequent formation of intra-subunit disulfide bond seems indeed unnecessary, despite that it might accomplish in a redox-modulated cytoplasm by external oxidative stress (*e*.*g*. PQ).

Furthermore, in view of the two-faced roles of CRP on oxidation deciphered herein, now it is of a certain degree to reconcile the controversy on CRP-engaged anti-inflammatory and pro-inflammatory activities previously with an interpretation of topological dependence^55^, and it becomes clear that both opposite effects of CRP on inflammation are likely more or less correlated with its solubility or physic status (or topological localization), *i*.*e*. soluble or insoluble. Only the soluble or free form of CRP (*e.g.* pCRP, pCRP*, mCRP monomer) having exposed binding sites can sequester reactive OxPCs and shield the active oxidation sites to avoid their lateral movement within membranes, thus emanating an antioxidative/anti-inflammatory role likely under controllable conditions^22^. However, this effect often seems transient in acute inflammations and instantly masked by the following pro-inflammatory actions through complement activation, during CRP structural dissociation^24^. Along with more and more mCRP converted and deposited on the membranes of tissues, the resulted mCRP aggregates become another form of pro-oxidative donors, triggering excessive production of ROS and aggravation of the inflammatory response, *i*.*e*. exhibiting a pro-inflammatory role independent of the complement system, as observed herein but overlooked previously. This notion should be an important supplement to discerning CRP’s functions except its signalling role in the innate immunity, and might provide a new insight for understanding the pathologic mechanisms of CRP-involved inflammations and diseases^6,7,9^. Actually, many studies have indicated that mCRP is more pro-inflammatory and pathology-associated^14,17,26^, as did Aβ42 peptide in AD^51,52^. The oxidative stress induced by mCRP aggregates deposited on membranes may considerably contribute to atherosclerotic and amyloid plaques and AMD drusen^7,9,14^. Therefore, given that CRP becomes recalcitrant to aggregation by fusing with hyper-acidic minipeptides (*e*.*g*. TUA2, ATS), these modified CRP might exert a more stable even predominant antioxidative/anti-inflammatory activity by restraining its pro-oxidative/pro-inflammatory role. Moreover, considering that protein can be prevented from aggregation by interacting with its hyper-acidic fusion cognates^56^, the interior mCRP might be of less chance to form aggregates owing to the *trans-*acting interference if introducing its hyper-acidified derivatives, and hence of less risk to induce oxidative stress and promote inflammation. Potentially, this concept and our results might be useful for developing new pharmaceutical therapies for those CRP-implicated inflammations and diseases^17,19,57,58^.

Finally, with respect to protein solubility improvement, fusion strategy has already been proven effectual for many proteins inherently prone to aggregation, and dozens of solubility-enhancers, as fusion partners or tags, were successfully applied^59,60^, including a few of hyper-acidic minipeptides^61–64^. Among them, TUA2, ATS are the short C-terminal acidic tails of chaperone-like tubulin in Arabidopsis^65^ and α-Synuclein in human^66^, respectively. Both hyper-acidic minipeptides can efficiently *in cis* improve the solubility and stability of many target proteins, and prevent them from aggregation and activity loss induced by environmental stresses, as reported in our previous studies and elsewhere^61,62,66^. Herein, they were also consistently capable to enhance the solubility of CRP as N-terminal fusion partners (Fig. 2C,D), and ensure even benefit its intrinsic antioxidative activity (Figs 1 and 4–7; Supplementary Figs 4 and 5). Upon adding TUA2 or ATS, CRP was converted to hyper-acidified fusion forms (TUA2-CRP, ATS-CRP) with much more negative charges (Supplementary Table 1), and likely become difficult to interact for aggregation due to strengthened electrostatic repulsion between similarly charged fusion moieties, thus allowing sufficient time for correct folding to achieve an elevated solubility^63,67^. Prospectively, this strategy could be utilized for scale preparation of soluble CRP to meet its diverse uses, alternative to the previous approaches^27,41,68–70^. Meanwhile, hyper-acidic minipeptides TUA2, ATS might also take a great deal of application potential in protein engineering and further genetic engineering.

## Materials and Methods

### Gene amplification and vector construction

Human *CRP* gene was initially amplified by whole-blood genomic PCR^71^ with primers CRP-5XKN, CRP-Rv (Supplementary Table 2) and Phusion high-fidelity DNA polymerase (NEB), according to its genomic structure (Supplementary Fig. 8). The crude product was 100-fold diluted as DNA template for the second round of PCR with gene-specific primers CRP-5XKN, CRP-3Sc (Supplementary Table 2). Then, the purified *CRP* fragment (encoding the mature chain (18–224 aa) without N-terminal secretory signal sequence) was subcloned into pET30s by *Xba* I and *Sac* I digestions to generate *E*. *coli* expression vector pET(CRP). On its basis, pET(CRPm) was further constructed by introducing the CRP mutant (F66Y/E81K) unable to bind phosphocholine^12,47^ via SOE-PCR^72^ using primers CRPm-Fw, CRPm-Rv (Supplementary Table 2). Plasmid pET30s, an analogue of pET30a(+), was previously created from pET32a(+) (Novagen) by *Nde* I removal of *trxA* fragment and self-ligation.

Vector pET30s(CRP) was constructed by relocating the *CRP* gene from pET(CRP) into pET30s behind the *Nco* I site. Using primer pairs TUA2-5Nd/TUA2-3Nd and ATS-5Nd/ATS-3Nd (Supplementary Table 2), the DNA fragments of hyper-acidic minipeptides TUA, ATS were re-amplified from plasmids pET(JcAPX1-TUA2) and pET(JcAPX1-ATS)^61^, and then inserted into pET30s(CRP) at the unique *Nde* I site to generate *E*. *coli* fusion expression vectors pET30s(t-CRP), pET30s(a-CRP), respectively.

Similarly, yeast expression vector pYES2(CRP) and plant expression vector pBI(CRP) were constructed by subcloning the *CRP* gene from pET(CRP) into plasmid pYES2 (Invtrogen) and pBI121 (Clontech) via dual digestions with *Kpn* I/*Xho* I and *Xba* I/*Sac* I, respectively. Using primer pairs TUA2-5Kn/TUA2-3Kn and ATS-5Kn/ATS-3Kn (Supplementary Table 2), the DNA fragments of TUA, ATS were re-amplified from pET(JcAPX1-TUA2) and pET(JcAPX1-ATS)^61^, and then inserted into pYES2(CRP) at the unique *Kpn* I site to generate yeast fusion expression vectors pYES2(t-CRP), pYES2(a-CRP), respectively. The same inserts were also introduced into pBI(CRP) at the unique *Kpn* I site to create plant fusion expression vectors pBI(t-CRP), pBI(a-CRP), respectively.

### Dot-plating test and spectrometric curve analysis of *E*. *coli* growth under oxidative stress

*E*. *coli* strain BL21(DE3) (Novagen) containing the recombinant vector pET(CRP), pET(CRPm), pET30s(CRP), pET30s(t-CRP), pET30s(a-CRP) or the empty vector pET30s, was grown in LB medium (+100 µg ampicillin mL^−1^) at 37 °C to (1) an OD_600_ of 0.6, then induced for 4 h at 37 °C with 0.5 mM isopropy-β-D-thiogalactoside (IPTG) (Sigma), or (2) directly to an OD_600_ of nearly 0.9 without induction. Subsequently, all bacterial cells were adjusted to an OD_600_ of 0.8, 2 µL of each serial dilution (1-, 2-, 4-, 8- fold) was dot-plated in a row on solid LB medium (containing 100 μg ampicillin mL^−1^ and 0.5 mM IPTG) with the final concentration of H_2_O_2_ (1.1 mM), PQ (0.5 mM) or CuSO_4_ (5 mM), and incubated at 37 °C for 1–2 d.

Meanwhile, bacterial cells of pET(CRP) and pET30s were initially cultivated at 37 °C to an OD_600_ of 0.4, then each supplemented with 0.5 mM IPTG and oxidative reagent (0.5 mM PQ or 5 mM CuSO_4_), and cultured at 37 °C continuously. The OD_600_ value of *E*. *coli* cells was measured every 1 h and plotted as the dynamic growth curve.

### Dot-plating test of *S*. *cerevisiae* growth under oxidative stress

*S*. *cerevisiae* strain INVSC1 containing the recombinant vector pYES2(CRP), pYES2(t-CRP), pYES2(a-CRP) or the empty vector pYES2, was grown at 30 °C in YPD medium (1% yeast extract, 2% peptone, 2% dextrose) to (1) an OD_600_ of 0.4, then precipitated, resuspended and induced in YPG medium (1% yeast extract, 2% peptone, 2% galactose) for 24 h at 30 °C, or (2) directly to an OD_600_ of nearly 1.9 without induction. Subsequently, all yeast cells were adjusted to an OD_600_ of 1.8, 2 µL of each serial dilution (1-, 10-, 100-, 1000-, 10000-fold) was dot-plated in a row on solid YPG medium with the final concentration of H_2_O_2_ (30 mM), PQ (0.2 mM) or CuSO_4_ (4 mM), and incubated at 30 °C for 2–4 d.

### Recombinant protein expression in *E*. *coli* and solubility determination

*E*. *coli* strain BL21(DE3) harboring any constructed prokaryotic expression vector was grown to an OD_600_ of 0.6 in LB medium (+100 µg ampicillin mL^−1^) at 37 °C, then induced for 4 h at 37 °C by 0.5 mM IPTG. Bacterial cells were harvested from a 14 mL culture by centrifugation, resuspended in 4 mL of 100 mM PBS buffer (pH 7.4), and lysed by ultrasonification. Aliquots (each 16 µL) of crude cell lysates (T) were separately fractioned into the supernatant (S) and pellet (P) by centrifugation. Meanwhile, 200 µL cell culture harvested before induction was precipitated and resuspended in 16 µL PBS buffer as the uninduced sample (UI). Subsequently, each sample was mixed with 4 µL 5× protein loading buffer, boiled for 5 min, and then subjected for 12% SDS-PAGE. The solubility of recombinant proteins on gels were estimated by band grey-densitometry via the program “Quantity One” (Bio-Rad).

### Generation of transgenic tobacco plants

*Agrobacterium tumefaciens* strain LBA4404 carrying plant expression vector pBI(CRP), pBI(t-CRP), or pBI(a-CRP) was transformed to sterile tobacco explants by leaf disc infiltration and kanamycin selection. Positive transgenic tobacco lines (briefly termed CRP, t-CRP, a-CRP) were strictly identified by multiplex genomic PCR with reciprocally combinatorial primers derived from *CRP*, fusion partner *TUA2*/*ATS*, and the backbone of vector pBI121 (Supplementary Table 2), and additionally confirmed by RT-PCR with specific primers of the introduced gene fragments.

### Leaf chlorosis test under oxidative stress and chlorophyll content determination

Leaf discs from WT and transgenic tobacco plants (T_0_ generation) cultivated in greenhouse for 4 weeks were submerged into 20 µM PQ solution or deionized H_2_O (CK), and placed in a growth chamber (26 °C, 70% humidity, 1500 Lux, a regime of 16 h light/8 h dark) for 7 d. After photo-recording, all leaf discs of each sample were collected and measured for the total chlorophyll content (mg·g^−1^ FW), as previously described^73^.

### Seed germination test under oxidative stress

Surface-sterilized seeds of WT and transgenic tobaccos were sowed on MS–agar medium containing 0.2 μM or 20 μM PQ, and placed in aforementioned growth chamber for 10 d. After photo-recording, the germination rate and seedling stem-length were determined respectively.

### Leaf-spray test under oxidative stress

Small plants of WT and transgenic tobaccos (growing in greenhouse for 1 week after a month of seedling cultivation in sterile Magenta box) with nearly identical growth status, were daily leaf-sprayed with 20 µM PQ solution for 7 d or 30 µM PQ solution for 3 d, using deionized H_2_O to spray as CK. After photo-recording, leaves (20 µM PQ-sprayed and CK) were sampled for histochemical staining, physiological assay and heterologous gene expression analysis as follows.

### Leaf histochemical staining

Intact tobacco leaves were *in situ* detected for dead cells by Trypan Blue staining^74^, for H_2_O_2_ by DAB staining^75^, and for O_2_^−^ by NBT staining^76^, respectively. In brief, the cleaned leaf samples were immersed into water-dissolved solutions containing 0.4% Trypan Blue, 0.1% DAB, or 0.2% NBT), and kept in dark for 3 min, then carefully washed with deionized water and subjected to chlorophyll clearance by boiling in anhydrous ethanol for 10 min.

### Oxidation-associated plant physiological assays

Protein contents of the leaf extracts were determined using BCA method, as previously described^77^.

MDA content was quantified by the thiobarbituric acid (TBA) reaction with minor modification of the method described before^78^. A 0.1 g leaf sample was homogenized in 1 mL 10% trichloroacetic acid (TCA). A 0.2 mL aliquot of the homogenate supernatant was mixed with 0.6 mL 0.6% TBA (in 10% TCA), incubated at 95 °C for 30 min and then immediately cooled in an ice-bath. After centrifuging at 10 000 g for 10 min, the absorbance of the supernatant at 532 nm was read and calibrated by subtracting the value for the non-specific absorption at 600 nm. The concentration of MDA (nmol mg^−1^ prot) was calculated using its extinction coefficient of 1.55 × 10^5^ M^−1^ cm^−1^.

Electrolyte leakage was determined according to a protocol described previously^79^. Ten leaf discs (1 cm^2^ each) were put into 25 mL deionized water in a test tube. After vacuuming and gentle agitation in a 25 °C water-bath for 2 h, the electrical conductivity (E_1_) was measured by a conductometer (Mettler Toledo). The same samples were boiled for 30 min, then returned to 25 °C water-bath for a gentle agitation of 2 h, and again recorded for their electrical conductivity (E_2_). The background value (E_0_) was determined in the control tube with deionized water only. Finally, the electrolyte leakage was calculated by the formula: (E_1_ − E_0_)/(E_2_ − E_0_) × 100.

The carbonyl content of oxidatively modified proteins was spectrometrically measured, as previously described^80^. Proteins were extracted from 0.1 g leaf tissue in 3 mL of 50 mM phosphate buffer (pH 7.4). The carbonyl groups on extracted proteins were reacted with 2,4-dinitrophenylhydrazine (Sigma), and the resultant hydrazone derivatives were estimated from the absorbance at 370 nm using a molar extinction coefficient of 2.2 × 10^4^ M^−1^ cm^−1^. Values (μmol mg^−1^ prot) were expressed as micromoles of carbonyl per milligram of leaf protein.

A modified method described before^81^ was used for H_2_O_2_ measurement. Leaf sample (0.1 g) was homogenized in 1 mL acetone. After centrifuging at 4 °C, 8 000 g for 10 min, all supernatant was transferred into a test tube, and sequentially mixed with 0.1 mL 5% TiSO_4_ and 0.2 mL ammonia. The yellow sediment by centrifuging at 4 000 g for 10 min was dissolved in 1 mL 2 M H_2_SO_4_, and subjected for recording the absorbance at 415 nm. H_2_O_2_ content (μmol/g FW) was obtained via standard calibration with a serial of H_2_O_2_ solutions of known concentrations.

CAT activity was determined by measuring the initial rate of H_2_O_2_ disappearance, as previously described^82^. Leaf sample (0.1 g) was homogenized in 1 mL 50 mM phosphate buffer (pH 7.0). After centrifuging at 4 °C, 10 000 g for 10 min, a 50 µL aliquot of the homogenate supernatant was added into the 2.95 mL reaction mixture containing 50 mM phosphate buffer (pH 7.0) and 15 mM H_2_O_2_. The decrease in H_2_O_2_ was recorded for 1 min as a decline in the absorbance at 240 nm. The activity of CAT (nmol/min/mg prot) was then calculated using a molar extinction coefficient of 4.36 × 10^4^ M^−1^ cm^−1^.

SOD activity was measured by the NBT reduction method described before^83^. Leaf sample (0.1 g) was homogenized in 1 mL 50 mM phosphate buffer (pH 7.8). After centrifuging at 4 °C, 10 000 g for 10 min, a 50 µL aliquot of the homogenate supernatant and 100 µL 0.1 mM riboflavin were sequentially added into the 2.85 mL reaction mixture containing 50 mM phosphate buffer (pH 7.8), 0.1 mM EDTA, 0.1 mM NBT, and 13.37 mM methionine. The absorbance at 560 nm of the sample and the parallel control were recorded after 30 min, and used for determining the inhibition rate and further calculating the activity of SOD (U/mg prot).

POD activity was assayed according to the guaiacol oxidation method, as previously described^84^. Briefly, leaf sample (0.1 g) was homogenized in 1 mL 50 mM phosphate buffer (pH 6.5). After centrifuging at 4 °C, 10 000 g for 10 min, a 50 µL aliquot of the homogenate supernatant was added into the 2.95 mL reaction mixture containing 50 mM phosphate buffer (pH 6.5), 2% H_2_O_2_, and 50 mM guaiacol. The increase of absorbance at 470 nm during 1 min was measured and used for determining the activity of POD (U/mg prot).

### Analyses of heterologous gene expression in transgenic tobaccos

Gene expression at mRNA level was examined by semi-quantitative RT-PCR. Briefly, total RNA from tobacco leaves were extracted with Trizol reagent (Invitrogen). By using random primers and M-MLV RTase (Promega), aliquots of RNA samples were reversely transcribed as the cDNA templates for PCR estimation of *CRP* transcriptional expression by primers CRP-iFw, CRP-iRv (Supplementary Table 2). Meanwhile, tobacco *18S rRNA* gene, as the internal gene expression reference, was analyzed with PCR primers Nt18S-iFw, Nt18S-iRv (Supplementary Table 2). Subsequently, the PCR bands of *CRP* and tobacco *18S rRNA* (419 bp, 552 bp, respectively) on gels were grey-densitometrically quantified by program “Quantity One”. The values of *CRP* were individually calibrated against the data of their coupled tobacco *18S rRNA*, in which the lowest ratio was further standardized as 1 to calculate the relative mRNA levels of CRP or its fusions expressed in transgenic tobaccos for comparison.

Gene expression at protein level was analyzed by Western blotting. Leaf sample (0.1 g) was homogenized in 400 µL protein extraction buffer (20 mM Tris·HCl (pH 7.5), 1 mM EGTA, 1 × protease inhibitor cocktail). After centrifuging at 4 °C, 10 000 g for 10 min, the supernatant was transferred to a new tube as the ‘S’ fraction, while the pellet was dissolved thoroughly in the same volume of 1% SDS (containing 1 × protease inhibitor cocktail) as the ‘P’ fraction. Aliquots of the ‘S’ samples (containing 20 μg protein each) and ‘P’ samples (using the same volume of its coupled ‘S’) were separated by 12% SDS-PAGE and then semi-dry transferred on PVDF membrane (Millipore). After blocking and washing, the membrane was sequentially reacted with CRP rabbit polyclonal antibody (ABP52927, Abbkine) at a dilution of 1:2000 and alkaline phosphatase (AP) conjugated goat anti-rabbit IgG (H + L) (A21120, Abbkine) as the secondary at a dilution of 1:5000, and then incubated into BCIP (5-Bromo-4-Chloro-3-Indolyl Phosphate)/NBT substrate mixture for color development. Meanwhile, the internal reference (actin) of tobacco cellular proteins was immuno-detected only on the ‘S’ fractions of total protein extracts, using anti-plant actin mouse monoclonal antibody (A01050, Abbkine) at a dilution of 1:2000 and AP-conjugated goat anti-mouse IgG (H + L) (A21110, Abbkine) as the secondary at a dilution of 1:5000. Finally, the membrane-bands of CRP or its fusions and tobacco actin were grey-densitometrically quantified by program “Quantity One”. The values (‘S’ + ‘P’) of CRP or its fusions were individually calibrated against the data of their coupled tobacco actin, in which the lowest ratio was further standardized as 1 to calculate the relative protein levels of CRP or its fusions expressed in transgenic tobaccos for comparison.

### Bioinformatic and statistical analyses

Primer design, vector construction, and basic analysis of protein features (*e*.*g*. size (aa), isoelectric point (*p*I), molecular weight (M.W.), and net charge at pH 7) were performed using the software, Vector NTI Suite 11.5 (Invitrogen). Experimental data statistics were processed using Microsoft Excel.

## Supplementary information

Supplementary Info file

## Data Availability

All data generated or analyzed during this study are included in this published article (and its Supplementary Information files).
