# Supplementary material for: Hyper-acidic fusion minipeptides escort the intrinsic antioxidative ability of the pattern recognition receptor CRP in non-animal organisms
Source: Sci Rep. 2019 Feb 28;9:3032. doi: 10.1038/s41598-019-39388-8 (PMC6395739; doi:10.1038/s41598-019-39388-8)
Supplement: Supplementary file 1 — Supplementary Info file [file 41598_2019_39388_MOESM1_ESM.pdf]

Supplementary Information

Title: Hyper-acidic fusion minipeptides escort the intrinsic antioxidative ability of the pattern recognition receptor CRP in non-animal organisms

Authors: Mengru Zhang[1], Yanjuan Liu[2], Zhibin Liu[1], Jianmei Wang[1], Ming Gong[2], Hu Ge[1], Xufeng Li[1], Yi Yang[1,*] & Zhurong Zou[2,*]

Supplementary Figures

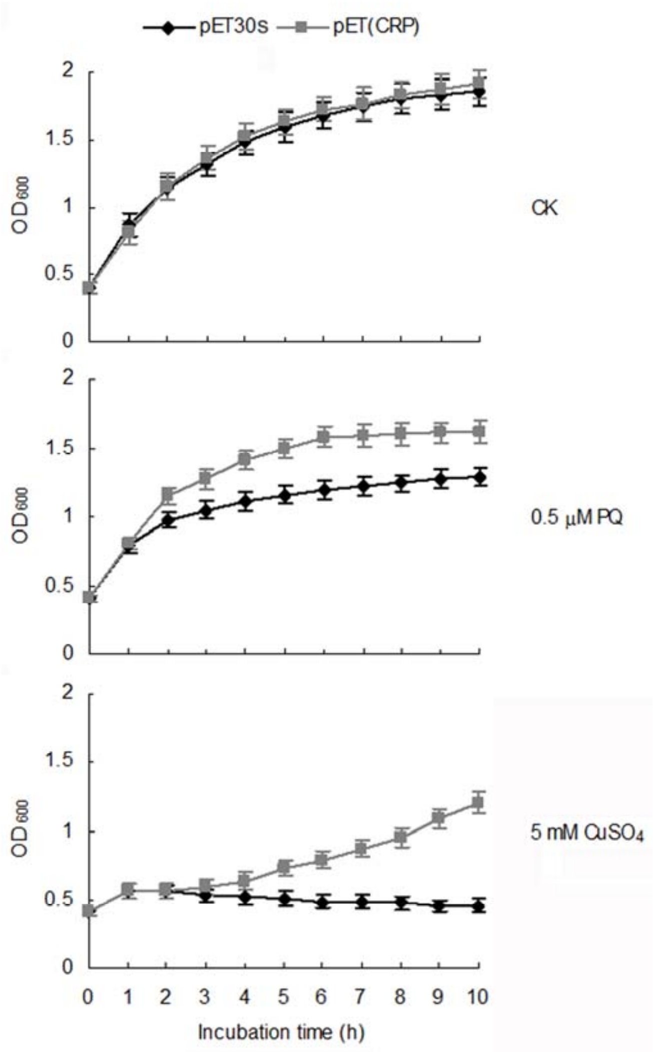

Supplementary Figure 1. *E. coli* growth curve comparison between the recombinant strain of pET(CRP) and the control strain of pET30s under normal condition (CK) and oxidative stresses of 0.5 mM PQ and 5 mM $CuSO_4$. Data are the means ± SD for three separate experiments.

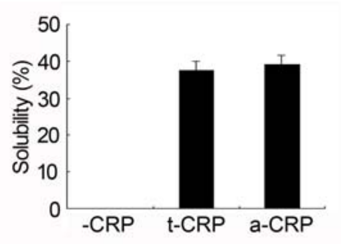

Supplementary Figure 2. Comparison of the solubility of various recombinant CRP proteins (-CRP, t-CRP, a-CRP) on SDS-PAGE gels (**Fig. 2**) determined by band grey-densitometric estimation. Data represent the means ± SD for three separate experiments.

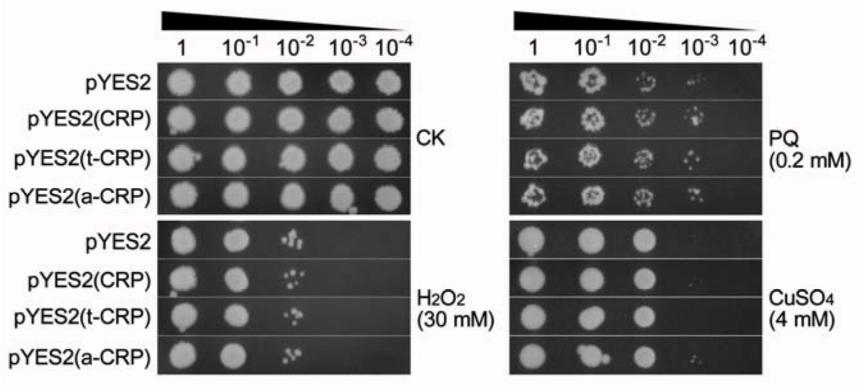

Supplementary Figure 3. Dot-plating test with serial dilutions (1-, 10-, 100-, 1000-, 10000-fold) to compare the colony growth of yeast cells without pre-induction among the recombinant strains of pYES2(CRP), pYES2(t-CRP), pYES2(a-CRP) and the control strain of pYES2 under normal condition (CK) and different oxidative stresses of 30 mM H_2O_2, 0.2 mM PQ, and 4 mM $CuSO_4$.

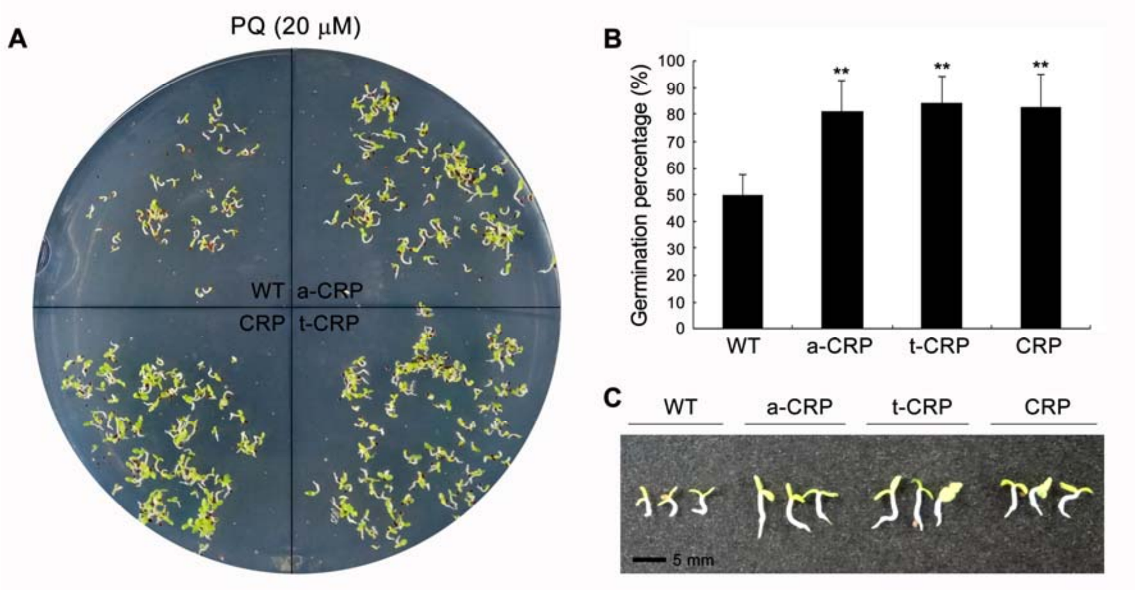

Supplementary Figure 4. Seed germination test of various CRP transgenic tobaccos under severe oxidative stress. Sterile seeds of WT and transgenic tobaccos (a-CRP, t-CRP, CRP) were germinated in a growth chamber on MS medium containing 20 μM PQ for 10 d, and then assessed for the germination profile (**A**), germination rate (**B**), and seedling status (**C**). Statistical significance was determined by Student's t test (n=3, >80 seeds per replicate experiment, data are the means ± SD, ** $P < 0.01$).

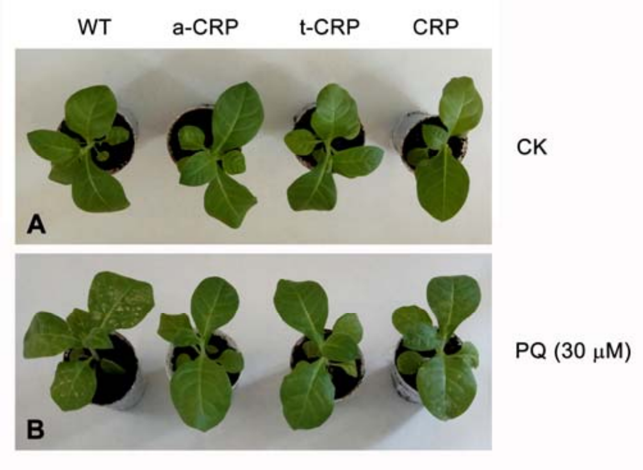

Supplementary Figure 5. Leaf spray test of various CRP transgenic tobaccos with a higher dose of oxidative treatment. Small plants (1-week growth in greenhouse after 1-month sterile cultivation of seedlings) of WT and transgenic tobaccos (a-CRP, t-CRP, CRP) were daily leaf-sprayed with deionized water (CK) (**A**) or 30 μM PQ solution (**B**) for 3 d. Then, the injury spots on leaves were assessed.

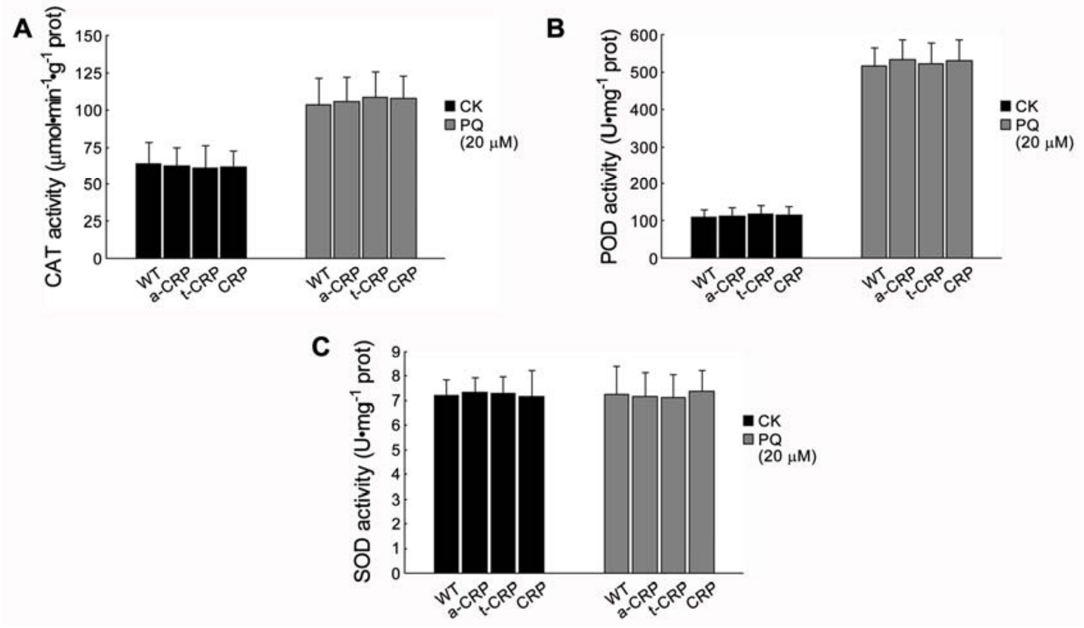

Supplementary Figure 6. Antioxidase activity changes in various CRP transgenic tobaccos after oxidative treatment. Leaves (daily sprayed with 20 μM PQ or deionized water (CK) for 7 d) of WT and transgenic tobaccos (a-CRP, t-CRP, CRP) were measured for the enzymatic activities of CAT (**A**), POD (**B**), and SOD (**C**). Statistical significance was determined by Student's t test (n=3, two replicates per experiment, data are the means ± SD). There was no significant difference between WT and transgenic tobaccos.

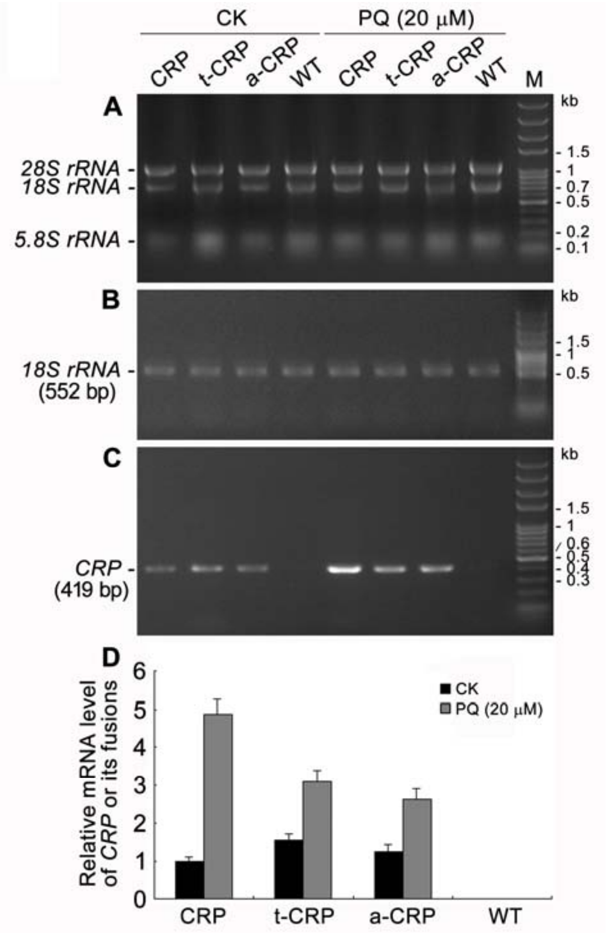

Supplementary Figure 7. Changes in the expression of CRP and its fusions at mRNA level in their transgenic tobaccos upon oxidative treatment. Leaves (daily sprayed with 20 μM PQ or deionized water (CK) for 7 d) of WT and transgenic tobaccos (CRP, t-CRP, a-CRP) were subjected for total RNA extraction (**A**) and subsequent semi-quantitative RT-PCR, using *18S rRNA* gene as the internal reference (**B**) to calibrate CRP transcriptional expression (**C**) in various transgenic tobaccos by gel-band grey densitometric estimation. The lowest ratio was standardized as 1 to obtain the relative mRNA levels of CRP and its fusions for comparison (**D**). Data represent the means ± SD for three separate experiments.

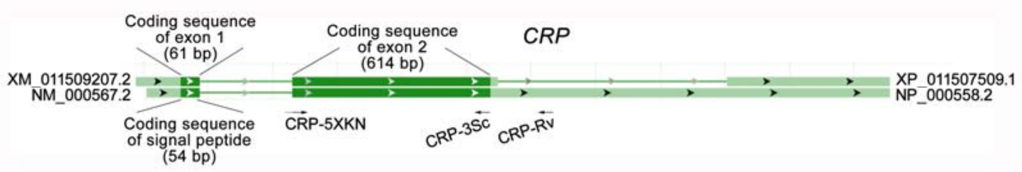

Supplementary Figure 8. The genomic structure of human *CRP* gene. The majority of CRP mature chain is encoded by its exon 2. Arrows indicate the primers used for *CRP* genomic amplification.

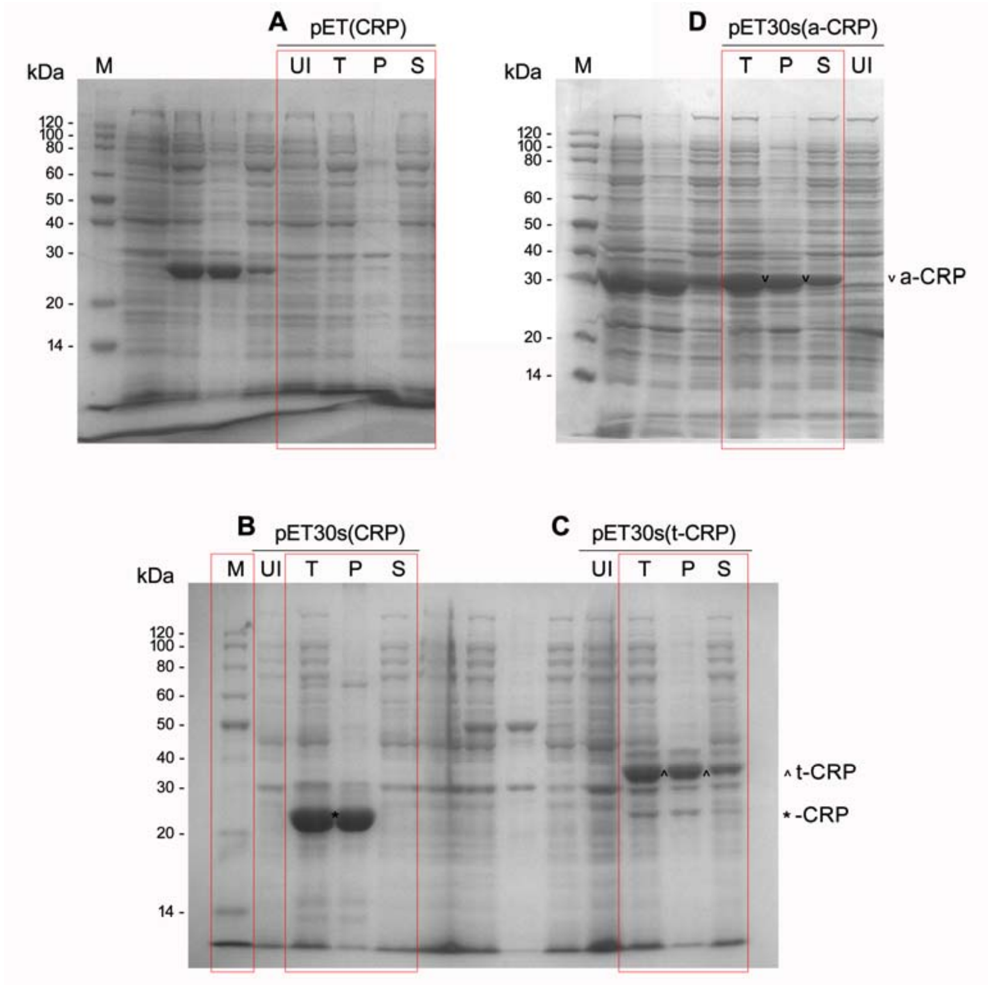

Supplementary Figure 9. Protein expression in *E. coli* of various CRP recombinant vectors including pET(CRP) (**A**), pET30s(CRP) (**B**), pET30s(t-CRP) (**C**) and pET30s(a-CRP) (**D**), analyzed by SDS-PAGE. M: protein molecular weight marker; UI: crude lysate of uninduced bacterial cells; T: crude lysate of induced bacterial cells; P, S: the pellet and supernatant fractions of induced bacterial cell lysate (T) after centrifugation, respectively. Red boxes indicate the constituents of **Fig. 2**.

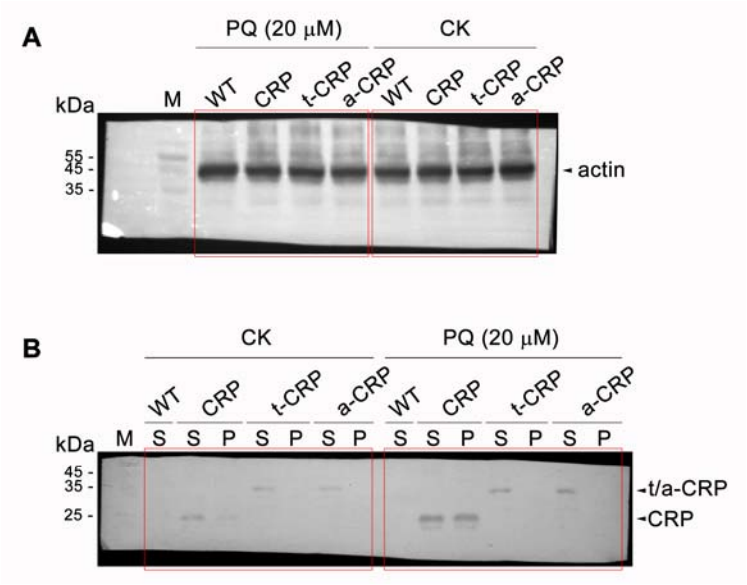

Supplementary Figure 10. Changes in the expression of CRP and its fusions at protein level in their transgenic tobaccos upon oxidative treatment. Leaves (daily sprayed with 20 μM PQ or deionized water (CK) for 7 d) of WT and transgenic tobaccos (CRP, t-CRP, a-CRP) were subjected for protein extraction plus fractionation into 'S', 'P' samples and subsequent immunoblotting with CRP polyclonal antibody. Actin ('S' fraction) was used as the internal reference (**A**) for calibrating the total expression ('S'+'P') of CRP protein or its fusions t/a-CRP (**B**) in various transgenic tobaccos. Red boxes indicate the constituents of **Fig. 8**.

Supplementary Tables

**Vector**	**Recombinant protein or fusion partner**	**Size (aa)**	**M.W. (kDa)**	***p*I**	**Net charge at pH 7.0**
pET(CRP)/ pYES2(CRP)/ pBI(CRP)	CRP	208	23.23	5.28	-4
pET30s(CRP)	-CRP [a]	252	28.09	5.71	-7
pET30s(t-CRP)	t-CRP [a]	295	32.84	4.67	-25
pET30s(a-CRP)	a-CRP [a]	297	33.17	4.77	-21
pYES2(t-CRP)/ pBI(t-CRP)	t-CRP [b]	257	28.55	4.34	-24
pYES2(a-CRP)/ pBI(a-CRP)	a-CRP [b]	260	28.93	4.40	-20
	TUA2 (t)	41	4.5	3.47	-18
	ATS (a)	43	4.83	3.36	-14

Supplementary Table 1. The properties of recombinant CRP and its fusion forms derived from their corresponding *E. coli*, yeast or plant expression vectors. [a] Symbol '-' indicates a short link peptide (44 aa) composed of His-tag, S-tag and the recognition site of enterokinase, which is encoded by the space sequence between *Nde* I and *Nco* I sites in plasmid pET30s. [b] Symbol '-' indicates a short link stretch of 7 aa, which is encoded by the space sequence between *Kpn* I and *Nco* I sites in the serial fusion expression vectors of the backbone pYES2 or pBI121.

Name	Sequence (5'→ 3') [a]	Remarks
CRP-5XKN	GGCTCTAGACGGTACCAGGAGACACCACCATGGGTCAGACAGATATGTCGAGGAAGGCTTTTGTG	Forward primer containing *Xba* I, *Kpn* I, and *Nco* I site as well as an integrated regulatory sequence suitable for CRP translation in *E. coli*, *S. cerevisiae* and plants
CRP-Rv	GACCAGACACTTTACCTCCATTCTC	Reverse primer downstream of CRP coding sequence (CDS)
CRP-3Sc	GTATCGAGCTCAGGGCCACAACTGGGGTTTGGTGAACAC	Reverse primer containing CRP stop codon and *Sac* I site
CRPm-Fw	GGATATAGGATACAGTTTTACAGTGGGTGGGTCTAAAATATTATTCGAGGTTCCTG	Forward primer for creating CRP mutant (F66Y/E81K)
CRPm-Rv	GTAAAACTGTATCCTATATCCTTAGACCAATATATGAGAATCTCATTGTCTTG	Reverse primer for creating CRP mutant (F66Y/E81K)
CRP-iFw	GCCTCTCAAAGCCTTCACTG	Forward primer inside the CDS of CRP
CRP-iRv	GTGACAGCACAAAGTCCCAC	Reverse primer inside the CDS of CRP
ATS-5Nd	GCTCGCATATGGATCAACTTGGTAAGAATGAA	Forward primer of ATS containing *Nde* I site
ATS-3Nd	GCGGACATATGAGCTTCAGGCTCGTAGTC	Reverse primer of ATS containing *Nde* I site
TUA2-5Nd	GTTGACATATGGGTGAGGGTATGGAAGAA	Forward primer of TUA2 containing *Nde* I site
TUA2-3Nd	GTGCTCATATGGTACTCCTCTCCTTCATC	Reverse primer of TUA2 containing *Nde* I site
ATS-5Kn	GTATTGGTACCGCAACCATGGGTGATCAACTTGGTAAGAATG	Forward primer of ATS containing *Kpn* I site
ATS-3Kn	GTCTTGGTACCTCAGCTTCAGGCTCGTAGTC	Reverse primer of ATS containing *Kpn* I site
TUA2-5Kn	GTATTGGTACCGCAACCATGGGTGAGGGTATGGAAGAA	Forward primer of TUA2 containing *Kpn* I site
TUA2-3Kn	GCGTTGGTACCTCGTACTCCTCTCCTTCATC	Reverse primer of TUA2 containing *Kpn* I site
Nt18S-iFw	GAAACGGCTACCACATCCAAG	Forward primer inside tobacco *18S rRNA* gene
Nt18S-iRv	GGCAAATGCTTTCGCAGTTG	Reverse primer inside tobacco *18S rRNA* gene

Supplementary Table 2. Primers used in this study. [a] The sites of introduced restriction enzymes are underlined.
